# Pathways Linking Exposure to Community Violence, Self-serving Cognitive Distortions and School Bullying Perpetration: A Three-Wave Study

**DOI:** 10.3390/ijerph17010188

**Published:** 2019-12-26

**Authors:** Mirella Dragone, Concetta Esposito, Grazia De Angelis, Gaetana Affuso, Dario Bacchini

**Affiliations:** 1 Department of Humanistic Studies, University of Naples “Federico II”, 80133 Napoli, Italy; concetta.esposito3@unina.it (C.E.); dario.bacchini@unina.it (D.B.); 2 Department of Psychology, University of Campania “Luigi Vanvitelli”, 81100 Caserta, Italy; grazia.deangelis2@gmail.com (G.D.A.); gaetana.affuso@unicampania.it (G.A.)

**Keywords:** adolescence, bullying perpetration, community violence exposure, morality, self-serving cognitive distortions

## Abstract

School bullying is a social phenomenon stemming from a complex interrelationship between the individuals and their environments. Underpinned by the social-ecological models, this study investigated the mediation of self-serving cognitive distortions (CDs) in the relationship between community violence exposure, as a victim and as a witness, and bullying perpetration. Bidirectional associations between violence exposure and bullying perpetration, and between CDs and bullying perpetration over time were also hypothesized. The study used a three-waves cross-lagged panel modeling in a sample of 829 Italian high school adolescents (46% males; *M_age_* [Time 1; T1] = 12.71; Standard deviation [SD] = 1.68). The results showed that being exposed to community violence as a witness at T1 increased the development of CDs at Time 2 (T2), which in turn promoted the bullying perpetration at Time 3 (T3). Being exposed to community violence as a victim was not a significant predictor of CDs and bullying perpetration over time. Bidirectional associations were found between witnessing violence and bullying perpetration, and between CDs and bullying perpetration. The association between community violence exposure and individual moral cognitions over time plays a crucial role in predicting bullying perpetration. These findings highlight the need to consider both contextual and individual factors in understanding and preventing bullying perpetration.

## 1. Introduction

There is broad consensus to consider bullying perpetration as a subtype of aggressive behavior that differs from the others because of its systematic nature and its manifestation in different patterns of interpersonal relationships [1], especially within the school context. Bullying behavior involves both direct (i.e., physical and verbal) and indirect (i.e., relational) forms of aggression and is marked by the deliberate intention to harm the other person and by an imbalance of psychological or physical power between the perpetrator and the victim [2,3]. Given the systematic nature of power’s abuse [4] to the detriment of weaker victims, school bullying has been considered a behavior of greater intrinsic moral relevance with respect to other aggressive behaviors [1,5,6].

Although the primary context in which bullying behavior occurs is school [3] and this manifestation is undeniably linked to personality characteristics [7], there is a recent call in the literature [8] for considering bullying as a social phenomenon stemming from a complex interrelationship between the individuals and the environments they inhabit, according to the more comprehensive framework represented by the social-ecological model [9,10]. Based on this framework [10], the purpose of the current study has been to investigated the longitudinal and simultaneous pathways linking child’s life experiences within the community (here represented by exposure to community violence as a victim and as a witness), individual moral cognitions (here represented by self-serving cognitive distortions - CDs according to the Gibbs’ model, [11]), with school bullying behavior.

To date, a large body of research on school bullying has mainly focused on the individual and their immediate systems, such as the family [12], the peer group [8] and the school context [13]. Only recently, studies focused on larger contextual factors have investigated specific aspects of the urban environment associated with school bullying, such as feeling of unsafety, poverty or gang affiliation within the neighborhood [14]. Generally, higher rates of bullying are found in communities in which violence is modeled and/or condoned, although the causal nature of these relationships remains unclear [14]. While there is a strong evidence that both exposure to community violence (e.g., [15,16,17]) and self-serving CDs [18,19] are associated with externalizing behaviors (i.e., aggression, conduct problems, delinquency), only a few studies (e.g., [20,21]), to our knowledge, have systematically examined how bullying behavior (as a specific subtype of aggression) is influenced by experiences of community violence exposure. Moreover, only one study [22] has investigated the associations of CDs, according to the Gibbs’ model, with the involvement in bullying behavior.

Finally, no study, to date, has investigated the role of exposure to violence within the neighborhood in predicting the development of self-serving CDs as intended in their moral dimensions, and the mediating role of such CDs in the relationship between community violence exposure and bullying perpetration. In this respect, most of the evidence comes from investigating the mediating role of acceptance of violence cognitions or biased social-information processing between exposure to community violence and aggressive behavior [23,24,25]; however, it is noteworthy that, to date, only a few studies have investigated the environmental precursors of moral attitudes underlying externalizing behavior in adolescence [26].

### 1.1. School Bullying in Adolescence

School bullying is one of the major social problems affecting children and adolescents in all parts of the world [8]. The developmental period during which these behaviors occur is the transition from childhood and primary school to adolescence and secondary school [27]. Adolescence is a life cycle stage characterized by greater psychosocial vulnerability [28] and has a critical role in terms of damages in several areas of adjustment and competence as relevant as self-esteem [29], academic engagement [30], school adjustment [31] and adolescent behavioral problems [32]. This decrease in psychosocial competences, as well as the increase in behavioral problems, seem to be related to the changes that occur in peer influence, which significantly increases during adolescence [30,33], putting youth at greater risk of involvement in deviant behaviors such as alcohol use and abuse [33] or bullying behaviors [34].

Consistent with the theoretical considerations discussed above about both the criticism of adolescence in terms of psychosocial vulnerability [28] and the most influential effects of peers during this period [30], previous longitudinal studies about the course of bullying along adolescence have found that the bullying trend is characterized by an initially increase as youths make the transition from elementary to middle school, peak after school transitions, and then gradually decrease during high school years [27,35,36]. This may be because youths have to re-establish their social status during the transition from primary to secondary school and, thus, bullying may be viewed as a deliberate strategy used to establish dominance as youths enter a new peer groups [27].

### 1.2. Exposure to Community Violence and Involvement in School Bullying Perpetration

The term “community violence” is generally defined and measured by researchers as instances of interpersonal harm or threats of harm within one’s neighborhood or community, and excludes related constructs such as domestic violence, physical maltreatment, sexual abuse, peer bullying, and media and video game violence [37]. Neighborhood structural characteristics, such as low income, concentrated disadvantage and unemployment, seem to play a prominent role in predicting youth exposure to violence within their communities [38].

Violence exposure within the community may be experienced both through a form of direct victimization and/or witnessing without being directly involved [39]. Most individuals are exposed to violence throughout their lives, but it is during adolescence that there is a dramatic peak in violence exposure [40,41], showing particularly stronger due to the increasing of environmental sensitivity during this life period [10].

Although children experience different kinds of violence in multiple contexts (i.e., the concept of “poly-victimization”; [42]), community violence seems to have a unique role in predicting a number of externalizing behaviors, such as aggression (e.g., [43]), antisocial behavior (e.g., [44]) and delinquency (e.g., [15]), accounting for other forms of violence exposure. Research examining the associations between community factors and school bullying has shown that low levels of neighborhood safety and access to guns and gang membership [14,45,46] predicted bullying perpetration. Nevertheless, to our knowledge, only a few studies have investigated the predicting role of community violence exposure on school bullying. Some studies, without distinguishing the differential effects of community violence witnessing and victimization, found a significant association between violence exposure within the community and bullying perpetration [21,47], over and above other socioenvironmental factors, such as poverty, inequality and political violence [48]. A seminal cross-sectional study by Schwartz and Proctor [49] found that children who had been a witness to or victim of community violence were more likely to bully their classmates. More in detail, witnessing community violence influenced school bullying through the mediation of socio-cognitive biases supporting positive evaluation of violent behavior.

Other studies have focused on the experience of witnessing or victimization. Using a latent transition mixture analysis, Davis et al. [20] reported that the largest proportion (25%) of youth who experienced heightened level of community violence as witnesses were more likely to be perpetrators of school bullying. Nonetheless, Andershed et al. [50] found that bullying others in school was related to a heightened risk of being violently victimized when out on the streets among both boys and girls.

Previous research corroborating the hypothesis that externalizing behavior could influence exposure to violence within the community over time [16,17] come from the literature on aggressive or delinquent behavior. More specifically, the results of these studies seem to confirm the bidirectional nature of these relations because the young people who engage in aggressive and delinquent behavior are more likely to put themselves in high-risk situations in which they are more likely to witness violence or to be victims of violence.

### 1.3. Self-Serving Cognitive Distortions and School Bullying Perpetration

Based on previous research on school bullying that found morality is a key factor for explaining perpetration [51], in the present study we will focus on the role of moral cognitions in mediating the association between exposure to community violence and bullying perpetration. In terms of such moral cognitive processes, the thinking patterns displayed by antisocial individuals are commonly referred to as “cognitive distortions,” a general umbrella term comprising a variety of theories consistent with a social-cognitive approach and constructs such as moral disengagement and social cognitive biases, that link behavior to the way one thinks about situations.

The concept of “cognitive distortions” has its origins in Sykes and Matza’s [52] theory of “neutralization,” which posits that individuals who act in an antisocial way try to resolve the discrepancy between their behavior and social norms by cognitive rationalization processes that deny or minimize the seriousness of their acts or justify them in some way [53]. According to Gibbs and colleagues’ theory [11], self-serving CDs are defined as “inaccurate or biased ways of attending to or conferring meaning upon experiences” [4] (p. 1). Barriga et al. [54] distinguish between primary and secondary self-serving CDs. Primary (i.e., *self-centered*) distortions serve as main motivators of aggressive behaviors because they are characterized by an egocentric bias which reflects more immature moral judgment stages stemming from self-centered attitudes, thoughts and beliefs (e.g., “*My idea in life is to satisfy myself to the extreme. I don’t need to defend my behavior. My thing is my thing. I don’t feel I am obligated to the world or to nobody*”; [55] p. 86).

Secondary distortions support the self-centered attitudes [11] and take the form of pre- or post-rationalizations serving to cognitively overcome dissonance between individual moral standards and behavioral transgressions and neutralizing potential empathy and guilt, thus avoiding damage to one’s self-image and facilitating deviant behaviors (e.g., “*Just because I shot a couple of state troopers doesn’t mean I’m a bad guy*”; [55] p. 172).

The often used four-category typology of self-serving cognitive distortions identifies four cognitive distortions: (i) *blaming others* (misattribution of blame for victimization or misfortune to innocent others); (ii) *minimizing/mislabeling* (antisocial behavior is depicted as not really harmful or even as an admirable outcome); and (iii) *assuming the worst* (gratuitous attribution of hostile intentions to others in a social situation; treating the worst scenario as inevitable; believing that improvement of one’s own or others’ behavior is impossible.

The longitudinal and reciprocal associations between moral cognition and behavior were investigated by Aquilar et al. [56], who found a reciprocal influence over time among values, moral judgment, considered similarly to CDs as a moral motivator [57] of externalizing behaviors (i.e., ‘aggression’, [58]; ‘bullying’, [59]) and antisocial behaviors, supporting that cognitions affect behavior, and behavior feeds back into cognitions, to reduce cognitive dissonance [11] when individuals became aware of discordance between their behavior and their beliefs.

Despite an increasing number of researchers [18,19,51] having found a link between self-serving CDs and externalizing behaviors, only one study [22], carried out with Australian adolescents, has examined the association between CDs and bullying at school. The authors found that bullies and bully-victims showed a higher tendency than victims and not-involved persons in assuming the worst, exhibiting minimizing-mislabeling and self-centered CDs, whereas only bullies were higher in blaming others. Overall, this research—which seems to confirm the role of distorted thinking patterns in the enactment of bullying behaviors—is consistent with a large body of studies developed within the theoretical framework of moral disengagement [60,61], finding such construct to be an important predictor of bullying [62].

### 1.4. Exposure to Community Violence, Self-serving Cognitive Distortions and School Bullying Perpetration

Despite the link postulated by social learning theory [63] and its crime-related extension [64] between chronic violent experiences that directly or indirectly expose children to antisocial models and the development of weaker internal moral standards, relatively few studies have investigated the association between neighborhood violence and morality [5,65]. The study by Wilkinson and Carr [66] tried to raise this point using qualitative data from male violent offenders, finding that individuals respond to exposure to violence, without distinguishing between violence witnessing or victimization, in many ways, some of which would be consistent with traditional concepts of moral disengagement. Bacchini et al. [67], for example, found that higher levels of exposure to community violence as a witness reduced the strength of moral criteria for judging moral violations. Other studies have found that being witness, but not victims, of community violence was associated with socio-cognitive biases in processing social situations [49]. These socio-cognitive biases take the form of hostile attributional bias, hostile social goals, and approval of aggression, thus influencing the individual’s ability to solve social problems and to correctly assess the negative consequences of their actions [25].

All these studies seem to be in line with the concept of “pathologic adaptation” to violence [68], according to which repeated exposure to violence in the community leads to a normalization of violence through mechanisms of moral neutralization, which in turn, facilitate the engagement in future episodes of violence. These results are also consistent with Anderson’s theorization of the “Code of the Street” [69]. As the author argued, living in neighborhoods where macrostructural patterns of disadvantage are radicalized lead to a sense of hopelessness and cynicism about societal rules and their application, thereby resulting in a street culture, the “Code of the Street”, that undermines mainstream conventional norms and shapes values that legitimize violence as an acceptable problem-solving tool among adolescents.

Finally, the depiction of moral cognitive processes as mediators of life experiences and as proximal mechanisms for externalizing behaviors is consistent with the biopsychosocial perspective on the development of adolescent conduct problems [10]. Based on this perspective, as a function of these aggressogenic life experiences, such as the repeated experience of being witness or victim of violence within the community, the child acquires knowledge and social-information-processing patterns that justify the appropriateness of behaving aggressively in problematic social situations, which in turn increase the likelihood to behave aggressively. Reciprocal influences among dispositions, contexts, and life experiences lead to recursive iterations across time that exacerbate or diminish the probability of aggressive behavior in social situations [10], such as the bullying behavior.

Taking this evidence as a starting point, further research is needed to clarify whether both experiences of violence exposure, as a victim and/or as a witness, are associated with constructs of moral cognitions such as CDs which, in turn, promote the involvement in school bullying behavior.

### 1.5. The Current Study

Based on the theoretical considerations discussed above, we investigated the role of CDs in mediating the relationship of community violence exposure, as a victim and as a witness, to school bullying perpetration in a longitudinal sample of Italian adolescents. We used a three-wave cross-lagged panel mediation design, which allowed us to control for baseline values of all variables in each wave and to examine the transactional nature and likely causal direction of the pathways linking exposure to community violence, CDs and school bullying perpetration. We expected that being exposed to violence within the community increased the likelihood that adolescents would develop self-serving CDs, which in turn, would promote the engagement in episodes of bullying perpetration. Specifically, we hypothesized significant associations between violence witnessing and both CDs and bullying perpetration, whereas no *a priori* hypotheses were formulated for violence victimization, due to the limited prior literature. Furthermore, consistent with our above-stated rationale, we hypothesized reciprocal associations between violence exposure and bullying perpetration, as well as between CDs and bullying perpetration over time, such that the more a person makes use of CDs, the more he or she is inclined to perpetrate bullying, and, *vice-versa*, the more a person is involved in bullying perpetration, the more he or she uses CDs to justify his or her immoral actions and maintain a positive image of him/herself.

Given that age- and gender-based differences have been observed in violence exposure, cognitive distortions and bullying behavior in previous research, we controlled the results for school grade and gender. Overall, prior studies have consistently found that males and older children are at greater risk for community violence exposure (e.g., [70]) and bullying perpetration (e.g., [71]); and males typically also self-report more cognitive distortions than females [22,72]. Parental socioeconomic status (SES) was also used as control variable, given its potential confounding effect on violence exposure [38] and bullying perpetration [73].

## 2. Materials and Methods

### 2.1. Participants and Procedure

The participants were part of a still ongoing longitudinal project that began in 2013. The study design originally involved sixth and ninth graders of two middle and three high schools in Arzano, a relatively small town located in the metropolitan area of Naples (Italy). This area is characterized by serious social problems such as high unemployment, school-dropout and the presence of organized crime ([74]). Overall, episodes of crime and violence such as robberies, menaces, extortions, presence of criminal organizations, trafficking and drugs possession, are raising a growing social alarm in the Italian context. According to the most recent data from Public Security Department of Italian Ministry of Interior (2018), the number of reported crimes in Italy is approximately 6600 per day. The metropolitan area of Naples is the second in Italy for reported crimes, with 568.91 every 100,000 inhabitants.

The sample for the current study consisted of 829 adolescents, 380 males and 449 females (46% males; 51% middle school students) assessed longitudinally from 2013 to 2015 (3 data points, 1 year intervals). The age of participants at T1 ranged from 11 to 16, with a mean age of 12.71 (*SD* = 1.68). Participants were from 10 different districts located in the metropolitan area of Naples, with the most part living in Arzano (79.7%). The districts were alike in terms of their housing density, residents’ family structure and SES.

Data collection took place every year in the spring. Parents’ written consent and adolescents’ assent were obtained prior to administration of questionnaires, which was conducted during classroom sessions by trained assistants. To reassure participants about reporting sensitive information and to encourage honest reporting, a complete guarantee of confidentiality was emphasized. Additionally, participants and their parents were informed about the voluntary nature of participation and their right to discontinue at any point without penalty.

### 2.2. Attrition Rate

The participation rate was approximately 85% across all time points, with 11% and 15% of T1 participants not assessed at T2 and Time 3 (T3), respectively (Total *N* = 125). At T2, participants were 332 males and 403 females (*N* = 735; Mean age of 13.57, *SD* = 1.59; 54.7% middle school students). At T3, participants were 321 males and 383 females (*N* = 704; Mean age of 14.49, *SD* = 1.58; 55.8% middle school students). Overall, we missed 15.5% and 14.7% of male and female participants, and approximately 7% and 24% of middle and high school students, respectively. The Little’s test [75] for data missing completely at random (MCAR) in SPSS 21 (IBM Corp.; Armonk, NY) was significant, *χ*^2^ = 368.033, *df* = 221; *p* ≤ 0.001, indicating that data were not missing completely at random. Subsequent *t*-test showed that participants who were missing at T2 and/or T3 significantly reported higher levels of CDs at T1 than participants who had data at all assessments (*p*s ≤ 0.05). Accordingly, full information maximum-likelihood (FIML) was used to handle missing data, enabling us to include all available data in the analyses. FIML does not estimate the missing data, rather it fits the covariance structure model directly to the observed and available raw data for each participant, offering unbiased estimates under the assumption that the missing data are missing at random [76].

### 2.3. Measures

#### 2.3.1. Exposure to Community Violence

Exposure to community violence was self-reported at each time point of the current study using two scales assessing community violence exposure through witnessing and victimization, respectively (Exposure to Community Violence Questionnaire; [16]). Items were selected from a review of the Community Experience Questionnaire by Schwartz and Proctor [49] on the base of their relevance to the specific urban context (for example, we did not ask how many times participants have been arrested or taken away by the police), and translated into Italian by two native Italian speakers, experts in psychology and fluent in English. Adolescents were asked to report violent incidents that had occurred during the last year and only serious real-life events from their neighborhoods and their communities, not incidents from movies or television or from day-to-day conflicts with other children at school. Each scale included six items, and adolescents were asked to report, using a 5-point scale (from *1 = never* to *5 = more than five times*), the frequency of their being the victim or witness of violence in the neighborhood during that time period. A sample item of being victimized was, “How many times have you been chased by gangs, other kids, or adults?”; a sample item for witnessing community violence was, “How many times have you seen somebody get robbed?”. Cronbach’s *α*s range from 0.79 to 0.88, and from 0.83 to 0.86, for violence victimization and witnessing, respectively.

#### 2.3.2. Self-Serving Cognitive Distortions (CDs)

Participants were asked to respond to the 39 items of the How I think Questionnaire (HIT; [54]; Italian validation by [77]), measuring self-serving CDs. Each item was rated on a 6-point Likert scale (from *1* = *agree strongly* to *6* = *disagree strongly*). Sample items were: “People need to be roughed up once in a while,” “Everybody breaks the law, it’s no big deal”. Cronbach’s *α* was 0.95 for T1 and 0.97 for T2 and T3.

#### 2.3.3. Bullying Perpetration

At each time point of the study, students were provided with a definition of bullying as intentional, repetitive aggressive behaviors including some sort of power imbalance between those involved, and were asked to indicate, using a 5-point scale (from *1 = never* to *5 = several times a week*), the frequency with which, since the beginning of the school year, they had exhibited eight different bullying behaviors, direct (i.e., physical, e.g., hitting/kicking, “I hit, kicked, or punched someone”, and verbal, e.g., threatening, “I threatened someone”) and indirect (e.g., excluding/ignoring, “I made nicknames for others that they didn’t like”). The questionnaire included eight items adapted from the bully-victim questionnaire [3]. Adaptation concerned: the extension of the number of items from seven to eight (the original item “I kept him or her out of things on purpose, excluded him or her from my group of friends or completely ignored him or her” was replaced by two distinct items “I kept him or her out of things on purpose” and “Excluded him or her from my group of friends or completely ignored him or her”); the response alternatives ‘sometimes’ and ‘now and then’ were replaced by the term ‘2 or 3 times a month.’ Cronbach’s *α*s were 0.86, 0.83 and 0.87, for T1, T2 and T3, respectively.

#### 2.3.4. Control Variables

Information about sociodemographic characteristics of the sample were collected asking participants to indicate their own age, sex and school grade. They were also asked to report both mothers’ and fathers’ educational level and occupational prestige. For educational level, the participants reported their parents’ level of educational attainment (from *1* = *finished only some primary classes or did not go to school* to *5* = *finished university or higher*). For parental occupational prestige, adolescents reported their mothers’ and fathers’ current job (from *1* = *has never worked outside the home for pay* to *10* = *professional*). Descriptive statistics are reported by neighborhood in Table 1. Using these four indicators, we created a composite score of SES by applying principal components analysis (PCA). The PCA model is consistent with a formative measurement in that the direction of causality goes from the items to the SES index and not vice versa [78].

### 2.4. Data Analysis

**Figure 1:**
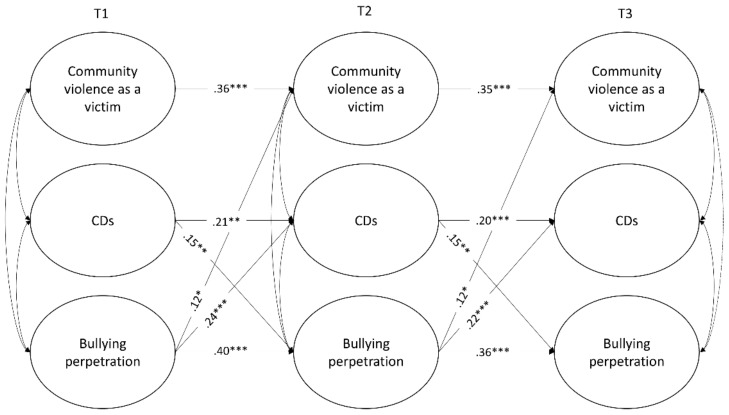
Cross-lagged mediational model with community violence exposure as a victim. CDs = Self-serving cognitive distortions. Reported coefficients refer to standardized estimates. For the sake of simplicity, nonsignificant paths, CDs random intercept and relations with control variables are omitted. The same results were obtained using the listwise deletion option. *p < 0.05, **p < 0.01, ***p < 0.001.

**Figure 2:**
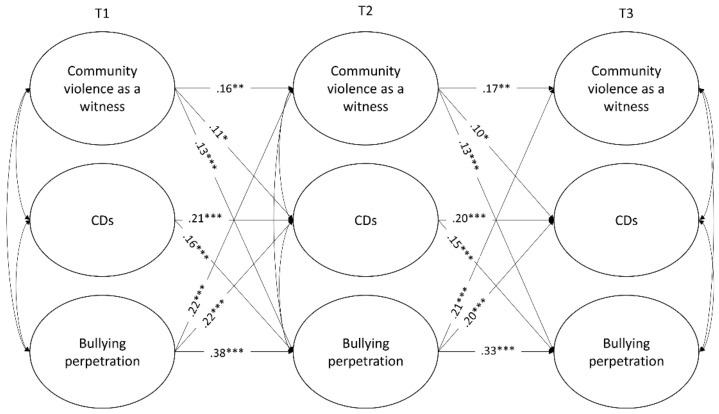
Cross-lagged mediational model with community violence exposure as a witness. CDs = Self-serving cognitive distortions. Reported coefficients refer to standardized estimates. For the sake of simplicity, nonsignificant paths, random intercepts of CDs and Community violence as a witness, and relations with control variables are omitted. The same results were obtained using the listwise deletion option. *p < 0.05, **p < 0.01, ***p < 0.001.

Three-wave cross-lagged panel analyses were used to test the hypothesized longitudinal relations among the study variables (Figure 1 and Figure 2). Extensive overviews of the use of this model for mediation analyses are given by Cole and Maxwell [79] and MacKinnon [80], as it allows to better investigate the likely direction of causal influence among variables, test for alternative models and lessen biases in testing mediation. The analyses were modeled in Mplus 8 [81] using the maximum likelihood estimation with robust estimators (MLR), due to the non-normality of violence exposure and bullying perpetration measures (skewness and kurtosis values ranged from 0.70 to 5.63 and 0.57 to 33.12, respectively). Missing data were handled by using full-information-maximum-likelihood (FIML) estimation of the parameters.

Based on the examination of intra-class correlation coefficients, suggesting that there were relatively stable individual differences over time in CDs and violence exposure as a witness (intra-class correlation coefficients were 0.44 and 0.46, respectively), two random intercepts that partial out the between-person stability of CDs and violence exposure as a witness were included in the analyses, such that the lagged coefficients represent within-person patterns of change [82].

Measures of violence exposure as a victim and bullying perpetration, for which the percentage of variance explained by differences between-persons was relatively low (intra-class correlations were ≤ 0.30), were included in the analyses as single indicator latent variables by using their relative composite mean scores as observed indicator, fixing the observed indicator’s factor loading to one and estimating the error terms from reliabilities [83]. Two models were run separately: one for exposure to community violence as a victim, the other for exposure to violence as a witness. The models included correlations among concurrent constructs at all time points, autoregressive paths for each construct across time, and all cross-lagged paths. Parental SES and adolescent school grade, and gender were included in the model as observed covariates.

Several indexes were used to evaluate the goodness of fit: The Yuan-Bentler [84] scaled chi-square statistic (YBχ^2^), the comparative fit index (CFI; [85]), the Tucker-Lewis index (TLI; [86]), and the root mean square error of approximation with associated 90% confidence intervals (RMSEA; [87]). A CFI ≥ 0.95 and RMSEA ≤ 0.06 indicate a model’s acceptable fit to the data [83]. To test equivalence of the structural parameters across time, two nested models were considered: a baseline model, in which parameters were freely estimated across time, and a fully constrained model, in which the structural paths were constrained to be equal over time. The Satorra–Bentler chi-square difference test (ΔSBχ2) was used to test relative fit of nested models [88]. When the more constrained model was rejected, a less restrictive model of partial invariance was tested in which, in accordance with modification indices, equality constraints on one or more parameters were relaxed until the change in fit was no longer significant. The mediated effects were tested using the bias-corrected bootstrap method, as recommended by Hayes and Scharkow [89], with 5000 bootstrap runs. Confidence intervals that do not contain the zero indicate significant indirect effects.

## 3. Results

### 3.1. Correlations

Correlations among study variables are shown in Table 2. Overall, statistically significant correlations among all study variables were found, both concurrently and longitudinally.

### 3.2. Cross-lagged Panel Modeling

#### 3.2.1. Exposure to Community Violence as a Victim

The model with all cross-lagged and autoregressive paths freely estimated showed an adequate fit to the data, YBχ2(8) = 20.59, *p* < 0.01; CFI = 0.99; RMSEA = 0.04, 90% C.I. [0.021, 0.067]. Imposing equality constraints to autoregressive and cross-lagged paths lead to a significantly worse of the model fit, ΔSBχ2(9) = 18.56; *p* < 0.05. In accordance with modification indices, the equality constraint on the path linking T1 violence victimization and T2 CDs was relaxed in order to improve the model fit, ΔSBχ2(8) = 13.34; *p* = 0.10; YBχ2 (16) = 33.50, *p* < 0.01; CFI = 0.98; RMSEA = 0.04, 90% C.I. [0.019, 0.054].

As can be observed in Figure 1, no significant associations were found between violence exposure as a victim and CDs. Bullying perpetration significantly predicted victimization, but not vice-versa. The cross-lagged and the mediation analyses confirmed the reciprocal associations between CDs and bullying perpetration over time, β = 0.04, t = 2.29, *p* < 0.05, 95% C.I. [0.011, 0.073] from T1 to T3 bullying perpetration through T2 CDs, and β = 0.03, t = 2.31, *p* < 0.05, 95% C.I. [0.011, 0.069] from T1 to T3 CDs through T2 bullying perpetration.

#### 3.2.2. Exposure to Community Violence as a Witness

The model with all cross-lagged and autoregressive paths freely estimated showed an adequate fit to the data, YBχ2(6) = 13.48, *p* < 0.05; CFI = 0.99; RMSEA = 0.04, 90% C.I. [0.009, 0.067]. Then, equality constraints were imposed to autoregressive and cross-lagged paths in order to test their invariance over time. Imposing them did not lead to a significantly worse of the model fit, ΔSBχ2(9) = 9.52; *p* = 0.39; YBχ2 (15) = 22.53, *p* = 0.09; CFI = 0.99; RMSEA = 0.03, 90% C.I. [0.000, 0.044]. As can be observed in Figure 2, being exposed to community violence as a witness significantly predicted CDs at each time point. Bidirectional relations between CDs and bullying perpetration, and violence exposure as a witness were also found. The mediation analysis highlighted a marginally significant indirect effect from T1 exposure to community violence as a witness to T3 bullying perpetration through T2 CDs, β = 0.02, t = 1.96, *p* = 0.05, 95% C.I. [0.002, 0.040], and a series of reciprocal associations between bullying perpetration and CDs. More specifically, bullying perpetration at T1 predicted bullying perpetration at T3 through the mediation of high CDs, β = 0.03, t = 2.39, *p* < 0.05, 95% C.I. [0.010, 0.067], whereas earlier high CDs increased CDs over time through the mediation of bullying perpetration, β = 0.03, t = 2.38, *p* < 0.05, 95% C.I. [0.010, 0.064].

#### 3.2.3. Control Variables

With respect to covariates, negative associations were found between school grade and violence exposure as a victim at T1 (β = −0.12, *p* < 0.001) and as a witness at T1 (β = −0.08, *p* < 0.05), with middle school students scoring higher than high school students. Furthermore, high school students reported higher levels of CDs at T2 (β = 0.11, *p* < 0.001) and T3 (β = 0.23, *p* < 0.001), and higher levels of bullying perpetration at T2 (β = 0.08, *p* < 0.05). Adolescent gender was negatively related to violence witnessing at T1 and T3 (βs = −0.17 and −0.15, *p* < 0.001, respectively), CDs at T1 and T2 (βs = −0.14 and −0.09, *ps* < 0.001 and 0.05, respectively), and bullying perpetration at T1 and T3 (βs = −0.24 and −0.15, *p* < 0.001, respectively), with males scoring higher than females. SES was negatively associated with CDs at T1 (β = −0.21, *p* < 0.001), T2 and T3 (βs = −0.07 and −0.08, *p* < 0.05).

## 4. Discussion

Using a three-wave cross-lagged panel design, the aim of the present study was to examine the pathways linking exposure to community violence, self-serving CDs and school bullying perpetration, hypothesizing that being exposed to violence within the community increase the likelihood that adolescents would develop self-serving CDs, which in turn would promote the engagement in episodes of bullying perpetration. More specifically, we expected significant associations between violence witnessing and both CDs and bullying perpetration, whereas no *a priori* hypotheses were formulated for violence victimization, due to the limited prior literature. Furthermore, we hypothesized reciprocal associations between violence exposure and bullying perpetration, as well as between CDs and bullying perpetration over time, such that the more a person makes use of CDs, the more he or she is inclined to perpetrate bullying, and, *vice-versa*, the more a person is involved in bullying perpetration, the more he or she uses CDs to justify his or her immoral actions and maintain a positive image of him/herself. All the effects were examined controlling for adolescent school grade, gender, and SES.

The findings showed that only violence exposure as a witness was associated to both bullying perpetration and CDs over time. More specifically, CDs mediated the association between witnessing violence and bullying perpetration. Bullying perpetration was a significant predictor of both violence witnessing and victimization; reciprocal associations between CDs and bullying perpetration were also confirmed.

The result that witnessing violence within the community predicted bullying perpetration corroborates previous studies finding that youth who experienced heightened levels of community violence as witnesses were more likely to be perpetrators of school bullying [20,49]. Similar findings have been found in the literature on deviant and antisocial behavior, indicating that witnessed violence provides behavioral models for such behavior, increases the tendency to believe that it is acceptable or even expected and desensitizes young people to the emotional effects of violence [17]. Differently, experiencing direct victimization had no longitudinal association with bullying. The lack of victimization effects on bullying perpetration may be explained by the fact that being victim of violence within the community could be linked with other variables that we did not include in the study, such as emotional dysregulation (as in the study by [49]) and internalizing rather than externalizing symptoms, as evidenced in the review by Fowler and colleagues [90]. Conversely, bullying perpetration significantly predicted violence exposure over time, both as a victim and as a witness, confirming that young people who engage in aggressive behavior such as bullying are more likely to put themselves in high-risk situations in which they are more likely to be witnesses or victims of violence [16,17,50].

Our result regarding the association between violence exposure as a witness, but not as a victim, and the individual tendency to make self-serving CDs over time is consistent with the biopsychosocial perspective [10] and in line with social learning theory [63,64], according to which adolescents who are exposed to violence within their living environments learn and internalize via observational learning a series of criminal/deviant models which take the form of social-cognitive schemas, beliefs and positive attitudes toward violence [91,92] that increase the probability that they will engage in future deviant behaviors [64]. This process, sometimes termed “cognitive desensitization to violence” [93], results in more approving violence beliefs, more positive moral evaluations of aggressive acts and more justification for inappropriate behavior inconsistent with social and individual’s moral norms. Despite existing studies [49] that have examined the development of biased cognitive processes through the youths’ experience of violence in their neighborhood, our study, that considers Gibbs and colleagues’ [94] theoretical formulation of CDs, extends prior findings by addressing this issue from a moral perspective. It also provides support for a “pathologic adaptation” model [68], according to which chronic exposure to community violence leads to a normalization of violence through the neutralization of moral standards.

Conversely, although we found positive concurrently correlations between violence exposure as victims and CDs, our results highlighted that victimization experiences did not have a contribution on self-serving CDs over time. These findings are consistent with the results of Schwartz and Proctor [49], showing that only being witness, but not victims, of community violence was associated with socio-cognitive biases supporting positive evaluation of violent behavior in processing social situations. Instead, the authors found that victimization experiences were associated with impairments in emotion regulation, in line with review by Fowler and colleagues [90].

Consistent with our expectations, we found that CDs promoted the engagement in bullying perpetration over time, and *vice-versa*. This finding provides support for social-cognitive approaches (e.g., [60,61]) that link behavior to the way one thinks about situations and confirms the results of previous studies (e.g., [62]) showing that youths need to construct attitudes and beliefs that justify their immoral actions in order to maintain a positive self-concept. As well, our findings strengthen the research on moral cognitions and externalizing problem behavior associations [19,22], extending the predictive role of CDs as conceptualized by Gibbs and colleagues [94], in explaining also peer-related aggression or bullying behavior, and not only serious delinquent acts such as antisocial or delinquent behavior [18,51]. Furthermore, the predictive role of bullying perpetration on CDs and the recursive association between cognition and behavior over time, such that cognitions affect behavior, and behavior feeds back into cognitions, is framed within the transactional developmental model [10] and provide support to the conceptualization of CDs as a form of post-rationalization serving to cognitively overcome dissonance between individual moral standards and behavioral transgressions and neutralizing potential empathy and guilt. Indeed, if on the one hand the tendency to make self-serving CDs is associated with bullying perpetration over time, on the other the more a person is involved in bullying perpetration, the more he or she uses CDs to reduce cognitive dissonance [11] and, thus, justify his or her immoral actions. Consistent to these findings are the results by Aquilar et al. [56] who found a reciprocal influence over time among values, moral judgment, considered similarly to CDs, and antisocial behaviors.

Finally, as hypothesized, the study highlighted a mediational pathway linking exposure to community violence as a witness, but not as a victim, to involvement in bullying perpetration through biased cognitive processes, although the magnitude of the effect was relatively modest. This result is consistent with previous similar research [49] and in line with Anderson’s [69] “Code of the Street” perspective, suggesting that living in neighborhoods where macrostructural patterns of disadvantage are radicalized facilitate the access to street’s subculture, that shapes pro-violence values such as the CDs to legitimatize the use of violence conceived as an acceptable problem-solving tool in neighborhoods where the street culture is widespread.

### 4.1. Limitations and Future Directions

This study has several limitations that need to be acknowledged. First, all the constructs are evaluated through self-report measures that may be subject to social desirability bias. Indeed, referring to the tendency to make self-serving CDs and to involvement in bullying behaviors, it is known (e.g., [95]) that adolescents are more careful about their social image than other age groups, and may be unlikely to report behavior that displays them in a negative light. Furthermore, regarding exposure to community violence, a more objective and comprehensive description of violence in the everyday lives of adolescents, including official data from national census agencies and police departments, may provide a more complete assessment of exposure to violence [96]. Future studies may benefit from utilizing a multi-informant approach (e.g., peer and teachers’ reports) jointly with self-report measures. Another limitation concerns the generalizability of the results, as the study included a sample from a limited geographical area in Southern Italy. Although most studies rely on a geographically-circumscribed sample, we are aware that multiple factors, including culture-specific beliefs and values, influence an individual’s cognitions and behaviors [44]. More research is needed to confirm that the explanatory model proposed in this study applies to populations from other, potentially different, cultural contexts. Furthermore, in this study we did not consider whether adolescents differently endorse CDs in response to situation-specific circumstances [97]. Future studies should investigate whether specific endorsements of CDs may differently mediate the relationship between violence exposure and bullying perpetration. Finally, taking account of the co-occurrence of different kinds of violence exposure from multiple contexts [42], further relevant variables should be considered, such as school and family-level risk predictors (e.g., school climate, school performance, peer influence, exposure to domestic violence) that could affect both the tendency to make self-serving CDs and involvement in bullying behaviors.

### 4.2. Prevention and Policy Implications

Notwithstanding these limitations, this study provides important suggestions for implementing appropriate interventions aiming at reducing adolescent involvement in school bullying perpetration. Overall, our findings confirm that prevention and intervention efforts should be focused on multiple ecological levels, including both contextual and individual factors. However, despite their effectiveness, such programs require economic resources that are not always available. As cognitive distortions have been found to play a crucial role on bullying perpetration in adolescents exposed to community violence, our study points to the benefit of school-based approaches that target the strengthening of children’s moral cognition, self-regulation and skills of social problem-solving. Such interventions would simultaneously influence school and community environments at a relatively low cost. The Equipping Youth to Help One Another (EQUIP) program [94] is an example of an effective cognitive-behavioral program developed within Gibbs’ theoretical framework aimed at educating young people at-risk or with behavioral problems in thinking and acting responsibly by decreasing individuals’ self-serving cognitive distortions, improving their social skills, and stimulating their moral judgment development using a peer-helping approach. Based on a positive peer culture, in which individuals feel responsible for each other and help one another, EQUIP is expected to have a great public impact given that it promotes, in the long-term, the development of a nonviolent and law-abiding culture, which represents the crucial condition for ensuring success in preventing and reducing children’s exposure to violence.

## 5. Conclusions

The present study provides further corroboration of the joint and reciprocal role of contextual and personal factors implicated in the enactment of school bullying behavior. Specifically, youths exposed to observational forms of violence in the neighborhood develop pro-aggressive moral cognitions taking the form of self-serving CDs. The internalization of these cognitive schemas about the world, along with the development of normative beliefs about violence, in turn, amplify the risk for involvement in bullying perpetration.

## Figures and Tables

**Table 1:** Proxy measures of socio-economic status (SES) by district. Reported coefficients refer to percentage rates.

Percentage of Adolescents Living in the District	District 1 3.1%	District 2 2.5%	District 3 1.8%	District 4 0.4%	District 5 1.6%	District 6 5.4%	District 7 2.3%	District 8 2.7%	District 9 0.6%	Arzano (District 10) 79.7%
*Mothers*										
*Educational Level*										
≤ Middle School	59.1	72.2	76.9	100	45.5	50.0	75.0	52.6	75.0	57.3
High School	27.3	22.2	7.7		36.4	34.2	6.3	31.6	25.0	26.4
≥ Bachelor’s Degree	9.1	5.6	15.4		18.1	2.6	6.3	5.3		10.3
Missing Data	4.5					13.2	12.4	10.5		6
										
*Occupational Status*										
Unemployed/Homemakers	59.1	77.8	84.6	66.7	54.5	52.6	81.3	52.6	75.0	63.9
Unskilled Worker (e.g., Factory Worker)	9.1		7.7	33.3		5.3	6.3	10.5	25.0	7.8
Skilled Worker (e.g., Employees, Teachers)	9.1	11.1			36.4	21.1	6.3	10.5		14.3
Professional or Managerial Worker	9.1					2.6				2.1
Missing Data	13.6	11.1	7.7		9.1	18.4	6.1	26.4		11.9
										
*Fathers*										
*Educational Level*										
≤ Middle School	59.1	72.2	69.2	66.7	36.4	60.5	81.3	68.4	75.0	64.4
High School	27.3	16.7	7.7	33.3	36.4	31.6	6.3	21.1		21.6
≥ Bachelor’s Degree	9.1	5.6	7.7		18.2			5.3	25.0	8.8
Missing Data	4.5	5.5	15.4		9	7.9	12.4	5.2		5.2
										
*Occupational Status*										
Unemployed	4.5	5.6	7.7	33.3	9.1	10.5	12.5	10.5		11.5
Unskilled Worker (e.g., Factory Worker)	27.3	44.4	53.8	66.7	36.4	39.5	50.0	42.1	25.0	36.6
Skilled Worker (e.g., Employees, Teachers)	27.3	16.7	7.7		27.3	21.1	12.5	15.8	25.0	25.1
Professional or Managerial Worker	13.6	11.1			9.1	7.9		5.3	25.0	4.1
Missing Data	27.3	22.2	30.8		18.1	21	25	26.3	25	22.7

**Table 2:** Correlations among study’s variables.

	1	2	3	4	5	6	7	8	9	10	11	12
1. T1 Community Violence Exposure as a Victim	1											
2. T1 Community Violence Exposure as a Witness	0.51***	1										
3. T1 CDs	0.23***	0.30***	1									
4. T1 Bullying Perpetration	0.20***	0.23***	0.30***	1								
5. T2 Community Violence Exposure as a Victim	0.26***	0.20***	0.08*	0.14***	1							
6. T2 Community Violence Exposure as a Witness	0.28***	0.48***	0.22***	0.22***	0.45***	1						
7. T2 CDs	0.14***	0.29***	0.50***	0.24***	0.18***	0.30***	1					
8. T2 Bullying Perpetration	0.18***	0.22***	0.27***	0.34***	0.19***	0.34***	0.34***	1				
9. T3 Community Violence Exposure as a Victim	0.27***	0.22***	0.16***	0.17***	0.33***	0.24***	0.21***	0.22***	1			
10. T3 Community Violence Exposure as a Witness	0.25***	0.40***	0.21***	0.24***	0.30***	0.49***	0.24***	0.26***	0.49***	1		
11. T3 CDs	0.11**	0.19***	0.38***	0.21***	0.16***	0.26***	0.46***	0.34***	0.25***	0.30***	1	
12. T3 Bullying Perpetration	0.12***	0.11**	0.21***	0.32***	0.16***	0.22***	0.27***	0.46***	0.20***	0.29***	0.43***	1

Note: CDs = self-serving cognitive distortions. *p < 0.05, **p < 0.01, ***p < 0.001.
